# Clinical and functional outcomes of locked plating vs. cerclage compression wiring for AO type C patellar fractures– a retrospective single-center cohort study

**DOI:** 10.1007/s00068-024-02633-5

**Published:** 2024-09-03

**Authors:** Steven Bickel, Kai Oliver Jensen, Felix Karl-Ludwig Klingebiel, Michel Paul Johan Teuben, Roman Pfeifer, Hans-Christoph Pape, Christian Hierholzer, Yannik Kalbas

**Affiliations:** 1 Department of Trauma Surgery, University Hospital Zurich, University of Zurich, Ramistr. 100, Zurich, 8091 Switzerland; 2 Harald-Tscherne Laboratory for Orthopaedic and Trauma Research, University Hospital Zurich, University of Zurich, Ramistr. 100, Zurich, 8091 Switzerland

**Keywords:** AO 34 C, Patellar fracture, Tension-band wiring, Compression cerclage wiring, Locked plating, Complications, Re-operations, Functional outcomes

## Abstract

**Purpose:**

Although “tension-band wiring” is still commonly used to stabilize patellar fractures, the technique has recently been scrutinized due to biomechanical insufficiency. Consequently, the AO Foundation renamed the principle to compression cerclage wiring (CCW). Several studies propose favorable outcomes when utilizing locked plating (LP). This study aims to compare outcome of CCW and LP for complex patellar fractures.

**Methods:**

A retrospective, single-center cohort study was performed on patients who underwent operative treatment for (AO 34 C-Type) patellar fractures between April 2013 and March 2023. Patients with a 12 month follow up were included. We grouped and compared patients based on the applied treatment strategy: group LP vs. group CCW. Primary outcome parameters included implant-related complications and revision surgeries. Secondary outcomes were length of stay, return to work and 12 months functional outcome (Lysholm score). Odd ratios for complications and revisions were calculated using the conditional Maximum Likelihood Estimate. The threshold for statistical significance was set at *p* < 0.05.

**Results:**

Of 145 patients, 63 could be included (group LP: *n* = 23 and group CCW: *n* = 40). Fractures in group LP were significantly more complex in regard to AO Classification (*p* < 0.001), number of fragments (*p* < 0.001) and degree of comminution (*p* < 0.001), yet odds of complications were significantly lower in group LP (OR: 0.147; 95%CI: 0.015–0.742; *p* = 0.009). K-wire migration was the most common complication in group CCW (20%). Odds of revision surgery were significantly lower in group LP (OR: 0.000; 95%CI: 0.000-1.120; *p* = 0.041). The average Lysholm score at one year was favorable in both groups (89.8; SD: 11.9 in group LP and 90.6; SD: 9.3 in group CCW; n.s.).

**Conclusion:**

In our study cohort, LP was routinely chosen for more complex fracture morphologies; nevertheless the data implies that LP may be considered as the superior fixation technique in regard to complications and revision operations. Especially, K-wire migration occurs frequently after CCW. The one year functional outcome was comparable between the groups, with both demonstrating good results. Prospective randomized studies are indicated to validate our findings.

## Purpose

As the largest sesamoid bone in the human body, the patella is of high biomechanical importance for the extensor mechanism of the leg. While patellar fractures are rather infrequent, constituting only 0.5–1.5% of all fractures, displaced transverse and comminuted fractures often require surgical intervention using open reduction and internal fixation, to restore the joint surface and extensor mechanism [1–4].

Tension-band wiring has been the most common technique to fix, especially transverse, patellar fractures [5–7]. Recent biomechanical studies, however, found evidence of an insufficient conversion of distraction- into compression forces when using this technique for patella and olecranon fractures [8–10]. Therefore, the AO Foundation has invalidated this principle in December 2023 and now promotes the term “cerclage compression wiring” (CCW) [11]. While this method is well-established for the fixation of patellar fractures, many implant complications, high reoperation rates and poor functional outcomes have been reported in literature [12–14]. In recent years, patellar plating, especially locked plating (LP), emerged as a promising alternative with good clinical outcomes and favorable biomechanical properties [15–19].

Direct comparisons of the (long-term) clinical outcomes between CCW and LP are rare in literature. Consequently, the aim of this study was to investigate the clinical outcomes of these two treatment options for patellar fractures, with the objective of testing two hypotheses:

1. Patellar fractures stabilized with LP exhibit a lower rate of complications and reoperations compared to those treated with CCW.
2. Patellar fractures treated with LP demonstrate superior functional outcomes one year postoperatively compared to CCW.

## Methods

The reporting of this retrospective cohort study was performed in strict adherence with the STROBE guidelines (STROBE: Strengthening the Reporting of Observational Studies in Epidemiology) [20].

### Setting

The study was conducted in accordance with the Declaration of Helsinki [21] and the Swiss Cantonal ethics committee (BASEC-Nrs. 2016_0188 and 2023_01704). The study site was the University Hospital Zurich, a Swiss Level I trauma center. Data collection and analysis were performed from December 2023 to March 2024. The period of recruitment was chosen from April 2013 to March 2023, to allow for a follow-up period of at least one year. All participants gave written approval for the use of retrospective data for scientific research.

### Participants

Every consecutive patient with a signed informed consent, who presented with a patella fracture during the recruitment period, has been screened for eligibility. Patients were identified through manual screening of operation records, aided by an automated search of our clinic information system for the corresponding ICD-10 Code (S82.0– Patella Fracture).

#### Inclusion criteria

encompassed primary operative treatment with LP or CCW for a patellar fracture. Further inclusion criteria were age ≥ 18 years, a complete dataset with a follow up ≥ 1 year and signed informed consent.

#### Exclusion criteria

were incomplete dataset / follow-up, repeatitive injuries of the same extremity and alternative fixation techniques.

### Study groups

Patients were stratified according to the implant utilized for patellar fracture fixation. ***group LP*** comprised patients who underwent primary operative treatment using locked plates. ***group CCW*** consisted of patients who received cerclage compression wiring. The allocation was not influenced by auxiliary screws or cerclages, however, patient were excluded if alternative principles (e.g. cannulated screws, trans-patellar sutures) were used.

### Rehabilitation protocol

By default, patients who underwent treatment with CCW were allowed full weight-bearing immediately after surgery. Patients treated with LP were restricted to 15 kg partial weight-bearing for the first two weeks after surgery, before transitioning to full weight-bearing. Both groups underwent a stepwise increase of knee flexion (30°/60°/90°) in 2 to 3 week intervals. Individual adjustments were made based on the surgeon’s assessment.

### Outcome parameters

**Fig. 1:**
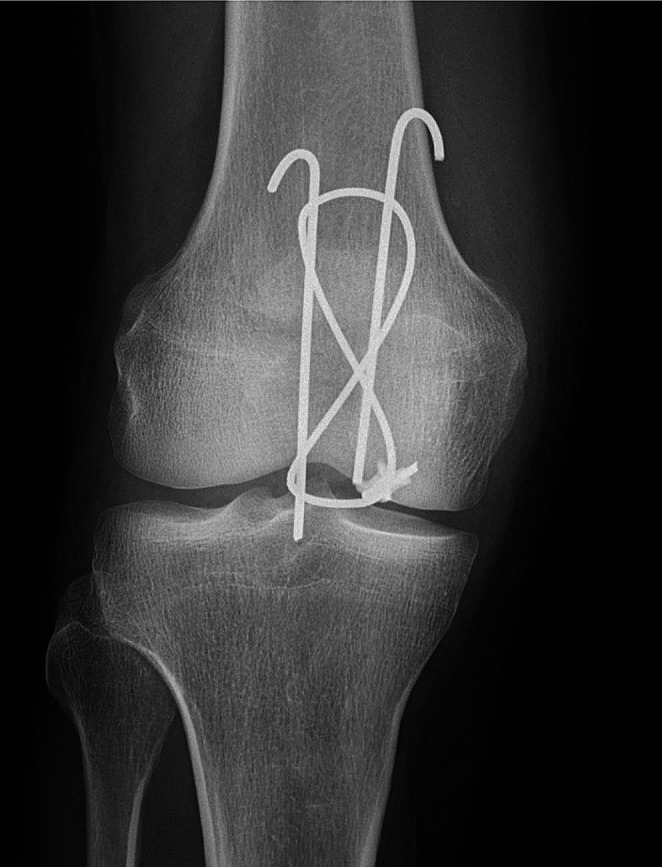
Demonstration of K-wire Migration

The primary outcomes of the study were the rate of complications and reoperations between the groups. Complications included infection, non-union, re-fracture, and implant complications. These included K-wire migration, wire breakage, skin perforation, and articular breach. (Fracture-related) infections were defined as described by the consensus statement of Metsemakers et al. [22]. Non-union was defined as a lack of any radiographic progression of bony consolidation for at least three months. Re-fracture was defined as any atraumatic fracture occurring during the rehabilitation period. K-wire migration was detected radiographically by comparison of intraoperative fluoroscopy and postoperative X-rays (as demonstrated in Fig. 1). This did not necessarily correlate to clinical symptoms. An articular breach occurred with any part of the hardware irritating the joint surface, as assessed on postoperative imaging (e.g. perforation of a screw). Reoperations were stratified into revision surgeries (i.e. re-osteosynthesis) and early (premature) implant removals. Additionally, the length of hospital stay was documented.

Furthermore, we evaluated the functional outcomes between the two study groups. To ensure relevant results, the functional outcomes were not assessed for patients with relevant concomitant injuries (e.g. polytrauma, severe traumatic brain injury, fractures of the same extremity) as well as patients with inconclusive reports. For all remaining patients, the domains from the Lysholm score were assessed twelve months postoperatively using both, medical record of the scheduled outpatient clinic visits and physiotherapeutic reports [23–25]. Relevant deficits in Range of Motion (ROM) were quantified by calculating mean knee extension lag (defined as less than 0° of extension) and mean knee flexion lag (defined as less than 120° of flexion) [26].

Moreover, the time from surgery to return to work was documented.

Additional variables included general demographics, comorbidities, and risk factors including smoking, diabetes, osteoporosis, and other relevant conditions (i.e. manifest cardiovascular disease, neoplasia). The “ASA physical status classification system” was used to assess the patients’ health status [27]. A full list of these variables is provided in Table 1. The mechanism of injury was categorized as “low-“(e.g. fall from standing height) or “high-energy”.

**Table 1:** Overview over patient demographics and trauma mechanism

	Overall		Group CCW	Group LP	*p*.
*n* =	63		40	23	
**Demographics**					
Gender, female (%)	32 (50.8)		24 (60.0)	8 (34.8)	n.s.
Age, mean (SD)	52.81 (19.12)		55.27 (19.59)	48.52 (17.89)	n.s.
Age > 70, *n* (%)	15 (23.8)		11 (27.5)	4 (17.4)	n.s.
BMI, mean (SD)	23.61 (4.07)		22.87 (3.75)	24.89 (4.38)	n.s.
Comorbidities and risk factors, *n* (%)	42 (66.7)		26 (65.0)	16 (69.6)	n.s.
Smoking, *n* (%)	24 (38.1)		14 (35.0)	10 (43.5)	n.s.
Diabetes, *n* (%)	7 (11.1)		4 (10.0)	3 (13.0)	n.s.
Osteoporosis, *n* (%)	15 (23.8)		12 (30.0)	3 (13.0)	n.s.
Others, *n* (%)	10 (15.9)		8 (20.0)	2 (8.7)	n.s.
ASA, median [IQR]	2.00 [2.00, 3.00]		2.00 [1.00, 3.00]	3.00 [2.00, 3.00]	n.s.
**Trauma Mechanism**					
High energy, *n* (%)	33 (52.4)		17 (42.5)	16 (69.6)	n.s.
Mechanism					n.s.
Fall from standing height, *n* (%)	28 (44.4)		21 (52.5)	7 (30.4)	
High falls, *n* (%)	7 (11.1)		4 (10.0)	3 (13.0)	
Vehicular accidents, *n* (%)	25 (39.7)		13 (32.5)	12 (52.2)	
Others, *n* (%)	3 (4.8)		2 (5.0)	1 (4.3)	
Concomitant injuries					n.s.
None, *n* (%)	40 (63.5)		29 (72.5)	11 (47.8)	
Lower Extremities, *n* (%)	3 (4.8)		2 (5.0)	1 (4.3)	
Multiple / Polytrauma, *n* (%)	20 (31.7)		9 (22.5)	11 (47.8)	

Fractures were classified according to the AO Classification System [28]. The number of fracture fragments with a diameter greater than 8 mm was assessed and the degree of comminution was categorized as “low”, “intermediate,” or “high” based on preoperative and intraoperative imaging and reports. Open fractures were graded according to Gustilo and Anderson. Procedural data, such as the interval from injury to surgery, duration of surgery and supplemental fixation methods e.g. screws, equatorial wires and augmentation with McLaughlin cerclage were documented.

#### Data collection and statistical analyses

All data was retrospectively extracted from patient records and images in our clinical information system. Fracture morphology and radiographic outcomes were assessed based on pre-/ postoperative computed tomography (CT) scans. If no CT scan was acquired, pre-/ postoperative X-ray images, intraoperative fluoroscopy images, and operative notes were used to obtain all relevant information. Radiographic images were assessed using “IMPAX EE Review” (Agfa HealthCare GmbH). Intraoperative fluoroscopy images were accessed using the DICOM viewer “Synedra view professional” (Synedra information technologies GmbH). Continuous parametric data were presented as mean and standard deviation, and non-parametric data as median and IQR. Categorical variables were presented as numbers and percentages. Statistical analyses were performed in R [29]. Data was visually tested for normality using histograms. Unpaired student T-tests were used for parametric data. Non-parametric data was tested using Wilcoxon–Mann–Whitney tests. Binary categorical data was assessed using Fisher’s exact test and non-binary categorical data using chi-squared test with Yates´ correction for continuity. Odds ratios were calculated using the conditional Maximum Likelihood Estimate. The threshold for statistical significance was determined as a *p*-value of < 0.05. The “ggplot2” package and Microsoft Office were used for data-visualization.

#### Bias and study size

To minimize selection bias and to achieve the maximal available study size within the given study period, all consecutive eligible patients were included. While the treatment strategy may have been in-part influenced by injury-specific factors, we expected that LP would be chosen for more complex fractures. Therefore, we did not expect any relevant bias towards ***group LP*** but rather the opposite.

## Results

### Study participants

**Fig. 2:**
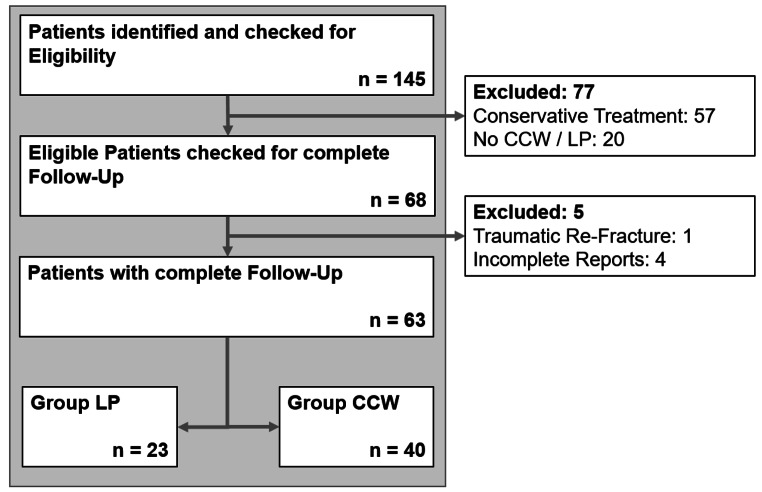
Flow chart illustrating the selection process

**Fig. 3:**
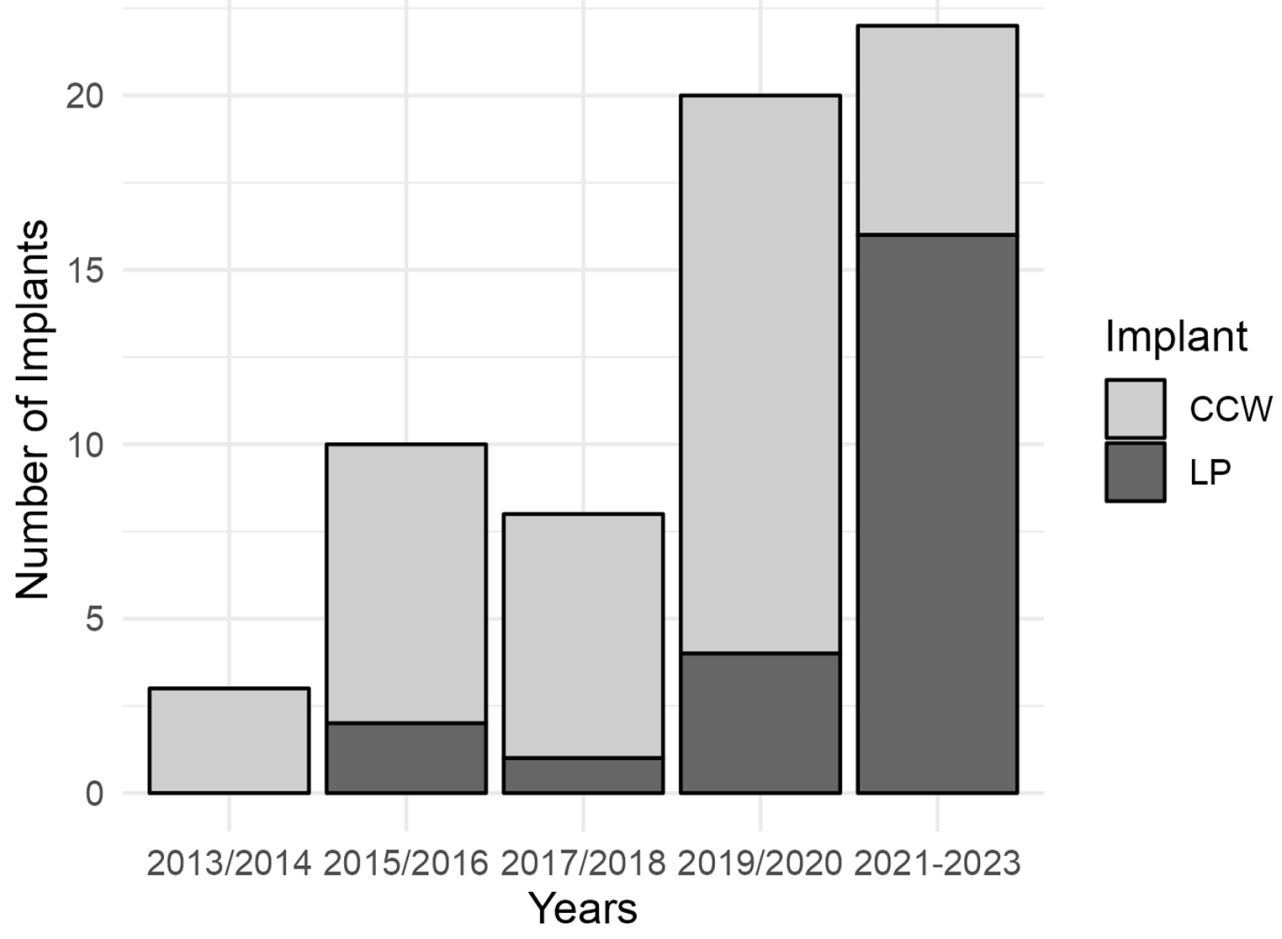
Implants by type over time

The patient identification and inclusion process is displayed in Fig. 2. We screened 145 consecutive patients who were treated for patellar fractures between April 2013 and March 2023. 77 patients were excluded due to non-operative treatment (*n* = 57) or an alternative fixation method (*n* = 20). An additional four patients had to be excluded due to incomplete reports. One patient had a second accident with a traumatic re-fracture two weeks after the initial surgery and was excluded as well. The remaining 63 patients were stratified into ***group LP*** (*n* = 23) and ***group CCW*** (*n* = 40). Figure 3 illustrates a timeline depicting the number of treatments with ***LP*** and ***CCW*** over five time periods. It becomes apparent that the use of locked plating increased within the last few years. Of the 23 plates used, six were Arthrex “Patella-SuturePlates”, DePuy Synthes’ “VA Locking Patella Plating System” was used 16 times, and one plate was a customized calcaneal plate.

### Patient demographics and trauma mechanism

Out of the 63 patients in the analysis, 32 were female and 31 were male. Of those, 15 patients were older than 70 years (***group LP***: *n* = 4, 17.4% vs. ***group CCW***: *n* = 11, 27.5%). The average age at injury was 52.8 years (SD: 19.1years). Table 1 provides an overview over patient demographics and trauma mechanism, indicating no significant differences among the study groups. However, we noted a higher proportion of male patients who underwent treatment with ***LP*** (65.2%) compared to ***CCW*** (40%). Furthermore, a greater percentage of ***group LP*** was affected by “high-energy” trauma (***group LP***: 69.6% vs. ***group CCW***: 42.5%), and had concomitant injuries (***group LP***: 52.2% vs. ***group CCW***: 27.5%).

### Fracture morphology and procedural data

Table 2 outlines fracture morphology and procedural data. Patellar fractures treated with ***LP*** were on average more complex and exhibited significantly higher AO Classifications (*p* < 0.001). While 91.3% of patients treated with ***LP*** presented with C3 fractures, more than half (55%) of patients treated with ***CCW*** had C1 fractures. We also noted a significantly higher number of fracture fragments in ***group LP*** (5.04, SD: 1.43 vs. 3.08, SD: 1.38, *p* < 0.001). ***group LP*** demonstrated a significantly higher degree of comminution (*p* < 0.001). Regarding procedural characteristics, no significant differences were noted in time to surgery (***group LP***: 3 days, SD: 3.6 days, ***group CCW***: 2.7 days, SD: 6.7 days) and duration of surgery (***group LP***: 113.5 min., SD: 47.9 min., ***group CCW***: 109.8 min., SD: 63.5 min.). A significantly larger percentage of supplemental fixation methods were used in the ***group LP*** (69.6%) compared to ***group CCW*** (32.5%, *p* = 0.01).

**Table 2:** Overview over fracture morphology and procedural data

	Overall		Group CCW	Group LP	*p*.
*n* =	63		40	23	
**Fracture morphology**					
AO/OTA Classification					**< 0.001**
C1.1, *n* (%)	14 (22.2)		14 (35.0)	0 (0.0)	
C1.2, *n* (%)	4 (6.3)		4 (10.0)	0 (0.0)	
C1.3, *n* (%)	4 (6.3)		4 (10.0)	0 (0.0)	
C2, *n* (%)	6 (9.5)		4 (10.0)	2 (8.7)	
C3, *n* (%)	35 (55.6)		14 (35.0)	21 (91.3)	
Number of fracture fragments, mean (SD)	3.79 (1.69)		3.08 (1.38)	5.04 (1.43)	**< 0.001**
Comminution					**< 0.001**
Low, *n* (%)	27 (42.9)		24 (60.0)	3 (13.0)	
Intermediate, *n* (%)	28 (44.4)		15 (37.5)	13 (56.5)	
High, *n* (%)	8 (12.7)		1 (2.5)	7 (30.4)	
Open fractures (Type II-III), *n* (%)	10 (15.9)		4 (10.0)	6 (26.1)	n.s.
**Procedural Data**					
Time to surgery (days), mean (SD)	2.83 (5.77)		2.72 (6.74)	3.00 (3.63)	n.s.
Duration of surgery (min.), mean (SD)	111.08 (58.12)		109.79 (63.51)	113.48 (47.90)	n.s.
Supplemental fixation methods, *n* (%)	29 (46.0)		13 (32.5)	16 (69.6)	**0.01**
Screws, *n* (%)	9 (14.3)		1 (2.5)	8 (34.8)	**0.002**
Equatorial wire, *n* (%)	17 (27.0)		12 (30.0)	5 (21.7)	n.s.
McLaughlin cerclage, *n* (%)	11 (17.5)		3 (7.5)	8 (34.8)	**0.016**

### Outcome variables

**Fig. 4:**
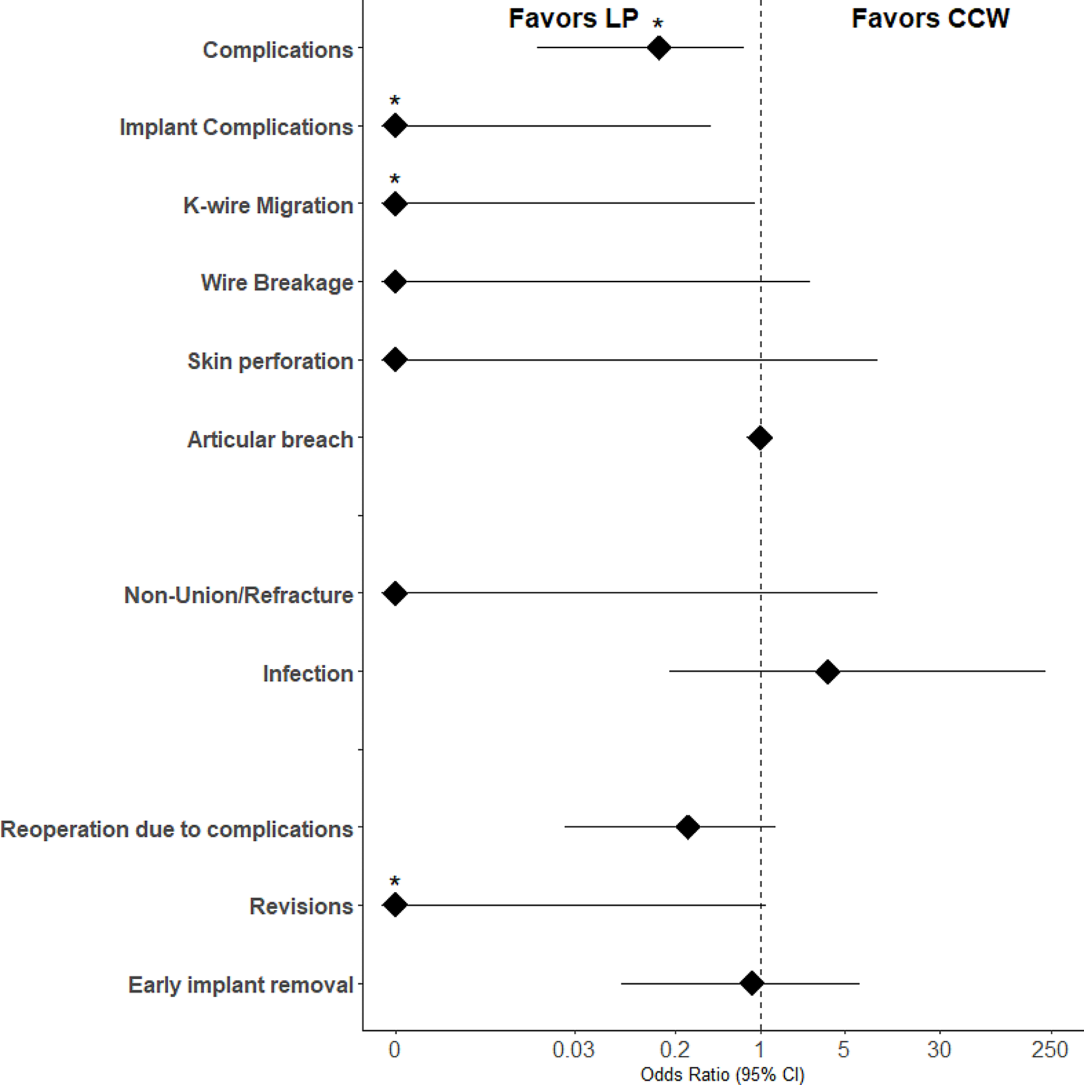
Forrest Plot illustrating Odds Ratios for Complications based on the Type of Fixation used

Outcomes and statistics are presented in Table 3***and*** Fig. 4. We noted significantly lower odds of complications in ***group LP*** (OR: 0.147; 95% CIs: 0.015 to 0.742; *p* = 0.009) compared to ***group CCW***. Among all patellar fractures treated with ***CCW***, 35% exhibited implant complications including K-wire migration, skin perforation, or wire breakage. K-wire migration was the most common complication and occurred in 20% of patients treated with ***CCW***. We did not note articular breaches or other implant complications when using ***LP***. Overall, significantly lower odds of implant complications in ***group LP*** were noted (OR: 0.000; 95% CIs: 0.000 to 0.397; *p* = 0.001). A comprehensive summary of the type of implant complication based on the AO Classification is provided in Table 4. Two patients in ***group LP*** and one patient in ***group CCW*** experienced postoperative infections. In ***group CCW***, one patient had a non-union and another suffered an atraumatic re-fracture during physiotherapy. Patients in the ***group LP*** underwent less reoperations (revision or premature implant removal) due to complications (LP: 8.7% vs. CCW: 27.5%), however this was not significant. Nevertheless, the odds for revision surgery were significantly lower in ***group LP*** (OR: 0.000; 95% CIs: 0.000 to 1.120; *p* = 0.041). The average length of hospital stay did not significantly differ between groups (***group LP***: 9.35 days, SD: 7.16 days, ***group CCW***: 9.7 days, SD: 10.13 days).

**Table 3:** Overview over rate of complications and reoperations and functional outcomes. Odds ratios and 95% confidence intervals (CI) were calculated for adverse outcomes when utilizing LP

	Group CCW	Group LP	Effect estimate (odds ratio)	Lower 95% CI	Upper 95% CI	*p*.
	40	23				
**Complications and reoperations**						
Complication, *n* (%)	16 (40.0)	2 (8.7)	0.147	0.015	0.742	**0.009**
Infection, *n* (%)	1 (2.5)	2 (8.7)	3.633	0.179	224.288	0.548
Non-Union or refracture, *n* (%)	2 (5.0)	0 (0.0)	0.000	0.000	9.296	0.529
Implant complications, *n* (%)	14 (35.0)	0 (0.0)	0.000	0.000	0.397	**0.001**
K-wire migration, *n* (%)	8 (20.0)	0 (0.0)	0.000	0.000	0.922	**0.023**
Wire breakage, *n* (%)	2 (5.0)	0 (0.0)	0.000	0.000	2.597	0.287
Skin perforation, *n* (%)	4 (10.0)	0 (0.0)	0.000	0.000	9.296	0.529
Articular breach, *n* (%)	0 (0.0)	0 (0.0)	NA	NA	NA	1
Reoperation of complication, *n* (%)	11 (27.5)	2 (8.7)	0.256	0.025	1.361	0.108
Revision, *n* (%)	7 (17.5)	0 (0.0)	0.000	0.000	1.120	**0.041**
Early implant removal, *n* (%)	4 (10.0)	2 (8.7)	0.859	0.072	6.600	1.000

	**Group CCW**	**Group LP**	**Difference of means**	**Lower 95% CI**	**Upper 95% CI**	**p.**
Length of hospital stay (days), mean (SD)	9.70 (10.13)	9.35 (7.16)	0.350	-4.031	4.735	0.631
**Functional outcome**						
Return to work (weeks), mean (SD)	18.08 (12.32)	22.58 (28.35)	-4.500	-24.114	15.103	0.732
Lysholm score, mean (SD)	90.58 (9.27)	89.83 (11.86)	0.750	-7.420	8.907	0.850
Flexion lag in °, mean (SD)	2,96 (9.02)	0 (0.0)	2.96	-0.60	6.53	0.099
Extension lag in °, mean (SD)	0 (0.0)	1.67 (5.77)	-1.67	-5.33	2.00	0.339

**Table 4:** Summary of the type of implant complication based on the AO classification

	C1	C2	C3
**Implant Complications**	**7**	**2**	**5**
Skin perforation	1	1	2
K-Wire migration	6	1	1
Wire breakage	0	0	2
Articular breach	0	0	0

On average, patients in ***group LP*** were able to return to work after 22.6 weeks (SD: 28.4 weeks), while patients in ***group CCW*** returned after 18.1 weeks (SD: 12.3 weeks). After one year, the average Lysholm score was 89.8 (SD: 11.9) in ***group LP*** and 90.6 (SD: 9.3) in ***group CCW***. The average flexion lag in ***group LP*** was 0° (SD: 0°) compared to 2.96° (SD: 9.01°) in ***group CCW***, while the average extension lag in ***group LP*** was 1.67° (SD: 5.57°) compared to 0° (SD: 0°) in ***group CCW***. None of these parameters were statistically significant.

## Discussion

Transverse and/or comminuted fractures of the patella typically necessitate open reduction and internal fixation to reconstruct the disrupted extensor mechanism and to anatomically reduce the joint surface. Locked plating has gained popularity in recent years with promising outcomes and favorable biomechanical properties [15–19]. Still, cerclage compression wiring remains a widely adopted technique [6, 12, 13]. In this retrospective cohort study, we compared the clinical and functional outcomes of locked plating to cerclage compression wiring in AO Type C patellar fractures. Our study identified the following key findings:

1. Locked plates are predominantly utilized for complex and highly comminuted fractures.
2. Despite the more complex fracture morphology, locked plates demonstrated a lower rate of complications, resulting in fewer revisions.
3. The functional outcomes one year postoperatively were comparable between the two study groups, with both surgical techniques demonstrating good results.

### Locked plates were used for more complex fracture morphology

The first finding aligns with the expected trend documented in existing literature. Particularly in cases of complex fracture morphology, CCW should be carefully considered and is often associated with unsatisfactory outcomes [7, 30–32]. In this study, patellar fractures treated with locked plates exhibited a significantly higher AO Classification, were divided into more fragments, and demonstrated a higher degree of comminution. This may account for the extensive use of supplemental fixation methods associated with plating. Auxiliary screws and equatorial wires were frequently used to stabilize single fragments that could not be adequately fixed with the primary implant.

The high number of inferior pole fractures in our cohort also explains the frequent use of McLaughlin cerclages. These challenging fractures can prove difficult to sufficiently reattach, especially when very comminuted. While both manufacturers offer plates with “legs” to fix these small fracture fragments at the lower pole, the additional utilization of a McLaughlin cerclage can provide subsidiary support of the osteosynthesis and is therefore commonly used in our practice.

Generally, locking plates have been increasingly utilized since 2019. This surge can be explained by an increased availability of specialized patella plates, a development driven by recent findings in the literature [15–19].

### Locked plates exhibit a lower rate of complications and revisions

The second major result highlights the significantly lower complication rate in patellar fractures treated with ***LP***. This is especially true for implant complications, which were identified in 35% of all patellar fractures treated with ***CCW***. These findings correspond well to the reported literature. Neumann-Langen MV et al. [33] demonstrated fewer postoperative complications with different plating systems compared with to CCW. Similarly, Meng D et al. [34] favored the X-shaped plating technique for its lower risk implant complications compared to two different CCW methods. Additionally, Tsotsolis et al. [35] conducted a systematic review of 18 studies, indicating a lower complication rate with plating compared to CCW based on data from existing literature. Nevertheless, these studies were often small cohorts or case series without comparable controls. The high rates of implant complications using CCW, particularly K-wire migration, wire breakage and skin perforation, has been demonstrated in various studies [12, 14, 36]. In our cohort, the higher rate of implant complications even resulted in a significantly higher rate of revision surgery.

Possible explanations for these outcomes include biomechanical limitations of this principle. Recent studies demonstrate a lack of conversion of distraction force into compression force when using the customary construct of two K-wires and a figure-of-eight cerclage-wire [8–10]. This led to the recent invalidation of this principle as a tension band by the AO Foundation, which now proposes the revised term cerclage compression wiring [11]. Accordingly, the previously recommended early loading to exert pressure on the fracture gap may be counterproductive and, in turn, could be a contributing factor to the high complication rate. In contrast, several studies have demonstrated that locked plates are biomechanically superior to CCW for the fixation of patellar fractures [10, 37, 38]. Another contributing factor to the high rate of implant complications associated with CCW may be the higher rate of potential user errors, especially regarding the precise placement of the K-Wires: These are supposed to be placed parallel and close to the articular surface. Further pitfalls may include choosing the correct length, sufficiently bending the wire and placing the wires deep behind the insertion of the tendon.

### Locked plates demonstrate comparable functional outcomes, despite complex fracture morphology

The interval from surgery to return to work, the average Lysholm score at the one-year follow-up and the deficits in ROM at the one-year follow-up were all comparable between the two study groups. Both average Lysholm scores indicated good functional outcomes falling within the range of 84 to 94 points [24]. This finding is promising, when considering that complex intraarticular fractures are typically associated with worse outcomes. Despite the more complex morphology, locked plating demonstrated comparable and very favorable functional outcomes one year postoperatively. These results also corresponded well with the scientific evidence. Meng et al. [34] reported favorable functional outcomes with both, the X-shaped plating technique and CCW fixation. Tsotsolis et al. [35] observed sufficient range of motion, low pain levels, and satisfactory functional outcomes with plating of patella fractures.

In a different study, Berninger et al. [39] observed a slightly higher incidence of limited ROM after LP compared to CCW after a minimum follow-up of 24 months. Our findings after a one-year follow-up did not show any relevant differences regarding ROM. The Lysholm scores showed comparable results in both studies.

Even though the functional outcomes were comparable at one year postoperatively, the rates of complications, especially implant complications, were significantly lower with locked plating. The resulting decrease in revision surgeries should be considered an important improvement of patient’s recovery. Given that the surgical approach, operative time, and required skill level were similar between both treatment methods, this raises the question of whether less complex fractures (i.e. C1) should also be treated with LP. This is further supported by the fact that implant complications in ***group CCW*** also frequently occurred in Type C1 fractures (see Table 4). However, economically, locked plates and screws are associated with higher cost, presenting an important counterargument. A cost-benefit calculation comparing implant prices to increased revision rates may be an important topic for future research. Additionally, the findings of this retrospective cohort study have yet to be confirmed by larger, preferably randomized, prospective studies.

### Strengths and limitations

This is a retrospective study and has associated limitations regarding potential individual and/or institutional biases. Besides this, there are two other important limitations, which need to be discussed. One is the necessity of an (generalized) informed consent, which is mandatory for the export of patient data from our clinic information system. This consent was introduced in 2014 and took several years until it was routinely obtained for all patients. This limitation can be observed in the constantly increasing number of patients being included (Fig. 3). A second limitation may be the more limited availability of patella plates before 2019, which can also be clearly observed in the increased usage of locked plating over the last years (Fig. 3). In this regard, there could have been a bias towards worse outcomes with CCW. Complex fractures may have been treated with insufficient fixation techniques, as the locked plates were not as readily available and not frequently used. Nevertheless, this would emphasize this study’s findings that LP has advantages compared to CCW, especially for complex fractures.

One the other hand, this study has several strengths, which we would like to point out. While our cohort is still relatively small, it represents one of the largest cohorts comparing CCW to LP to date. Clinical and functional outcomes were evaluated for a long follow-up period, identifying a comprehensive understanding of treatment effectiveness. Moreover, the study was conducted at a single center where CCW and LP are frequently utilized for stabilizing patellar fractures. Surgeons are adequately trained for both techniques, minimizing potential treatment variability and allowing for a direct comparison under comparable conditions.

In our cohort, a relevant proportion of patients suffering patellar fractures were either older than 70 years (23.8%) or were diagnosed with osteoporosis (23.8%). Both conditions are associated with poor bone quality, which can affect the healing process. Future research should specifically investigate treatment options for patellar fractures in geriatric and osteoporotic patients to better understand their outcomes.

## Conclusion

The current retrospective study demonstrates that, despite more complex fracture morphology, locked plates demonstrate a lower rate of complications, leading to fewer revisions compared with cerclage compression wiring. However, one-year functional outcome does not differ significantly between groups, with both demonstrating good results. Our data suggest that locked plating may be superior compared to cerclage compression wiring. Future prospective (randomized) studies are indicated to validate these findings.

## Data Availability

No datasets were generated or analysed during the current study.

## Abbreviations

BMI: Body Mass Index
CCW: Cerclage Compression Wiring
CT: Computer Tomography
LP: Locked Plating
ROM: Range Of Motion
